# Genetics and Epigenetics: Implications for the Life Course of Gestational Diabetes

**DOI:** 10.3390/ijms24076047

**Published:** 2023-03-23

**Authors:** William L. Lowe

**Affiliations:** Department of Medicine, Division of Endocrinology, Metabolism and Molecular Medicine, Northwestern University Feinberg School of Medicine, Rubloff 12, 420 E. Superior Street, Chicago, IL 60611, USA; wlowe@northwestern.edu; Tel.: +1-(312)503-2539

**Keywords:** gestational diabetes, genetics, epigenetics, DNA methylation, non-coding RNAs, type 2 diabetes, cardiovascular disease

## Abstract

Gestational diabetes (GDM) is one of the most common complications of pregnancy, affecting as many as one in six pregnancies. It is associated with both short- and long-term adverse outcomes for the mother and fetus and has important implications for the life course of affected women. Advances in genetics and epigenetics have not only provided new insight into the pathophysiology of GDM but have also provided new approaches to identify women at high risk for progression to postpartum cardiometabolic disease. GDM and type 2 diabetes share similarities in their pathophysiology, suggesting that they also share similarities in their genetic architecture. Candidate gene and genome-wide association studies have identified susceptibility genes that are shared between GDM and type 2 diabetes. Despite these similarities, a much greater effect size for *MTNR1B* in GDM compared to type 2 diabetes and association of *HKDC1*, which encodes a hexokinase, with GDM but not type 2 diabetes suggest some differences in the genetic architecture of GDM. Genetic risk scores have shown some efficacy in identifying women with a history of GDM who will progress to type 2 diabetes. The association of epigenetic changes, including DNA methylation and circulating microRNAs, with GDM has also been examined. Targeted and epigenome-wide approaches have been used to identify DNA methylation in circulating blood cells collected during early, mid-, and late pregnancy that is associated with GDM. DNA methylation in early pregnancy had some ability to identify women who progressed to GDM, while DNA methylation in blood collected at 26–30 weeks gestation improved upon the ability of clinical factors alone to identify women at risk for progression to abnormal glucose tolerance post-partum. Finally, circulating microRNAs and long non-coding RNAs that are present in early or mid-pregnancy and associated with GDM have been identified. MicroRNAs have also proven efficacious in predicting both the development of GDM as well as its long-term cardiometabolic complications. Studies performed to date have demonstrated the potential for genetic and epigenetic technologies to impact clinical care, although much remains to be done.

## 1. Introduction

Gestational diabetes mellitus (GDM) is defined as maternal hyperglycemia that is first recognized during pregnancy, excluding those with overt diabetes [1]. Approaches to screening for GDM vary, but universal screening for GDM at 24–28 weeks gestation has been widely adopted. The two primary approaches used for screening are a one-step approach that includes a 75 gm oral glucose tolerance test (OGTT) or a two-step approach that includes a glucose challenge test followed by a 100 gm OGTT in those whose glucose level exceeds 130 to 140 mg/dL during the glucose challenge test [1,2]. The diagnostic criteria used to diagnose diabetes vary, although a number of organizations, including the World Health Organization, have adopted criteria based on one-step screening proposed by the International Association of the Diabetes and Pregnancy Study Groups (IADPSG) [3]. The prevalence of GDM varies depending on the diagnostic criteria that are used and the population being screened, but the International Diabetes Federation has estimated that GDM occurs in as many as one in six pregnancies worldwide, with ~90% of cases occurring in low- and middle-income countries [1]. 

Several factors increase the risk for GDM, with a family history of type 2 diabetes (T2DM) and a history of GDM in a prior pregnancy being among the strongest risk factors [1]. Additional risk factors include maternal overweight and obesity and higher maternal age, while previous macrosomic birth, maternal hypertension, and greater gestational weight gain, especially early in pregnancy, are also associated with higher risk. Finally, ancestral background is an important risk factor, as the prevalence of GDM varies across ancestry groups with South and East Asian, Black, Native American, and Hispanic ancestries at higher risk compared to other groups [1].

Metabolic changes occur during pregnancy to address the nutritional needs of the growing fetus [1,4,5]. Prominent among these changes is a progressive increase in insulin resistance, which occurs largely during the second and third trimesters of pregnancy. This increase in insulin resistance is a result of the placental secretion of hormones, including growth hormone and human placental lactogen. Secretion of inflammatory mediators from the placenta and other tissues also contributes to insulin resistance in pregnancy [6,7]. To compensate for the increase in insulin resistance, insulin secretion is enhanced due to an expansion of beta cell mass [1,4]. The expansion of beta cell mass is regulated, at least in part, by prolactin and human placental lactogen. GDM results from a relative deficiency of insulin secretion, which is a result of inadequate insulin secretion to compensate for the increased insulin resistance [1,4]. Thus, mothers with baseline high insulin resistance and/or a pre-existing defect in beta cell function are at high risk for GDM. 

GDM is associated with a number of short-term maternal and fetal complications. These include a higher risk for large gestational age birth, shoulder dystocia, birth injury, neonatal hypoglycemia and respiratory distress, preterm delivery, cesarean section, gestational hypertension, and preeclampsia [1,4]. GDM also has important implications for the life course of affected women [1,2,4]. GDM is associated with a high risk of developing T2DM postpartum. Risk estimates ranging from 1.3 to 47.0 for progression from GDM to T2DM have been reported [8,9]. This variation in risk is related to ancestry group, nationality, duration of follow-up, and methods of diagnosing both GDM and T2DM [8]. A recent meta-analysis demonstrated a 10-fold higher risk for progression to T2DM in women with a history of GDM [10]. Risk factors for progression to T2DM include maternal age, pre- and postpartum BMI, family history of T2DM, need for insulin treatment of GDM, fasting glucose during pregnancy, and offspring sex from the GDM pregnancy [11,12,13,14,15,16,17,18,19]. The high risk for progression to T2DM likely reflects the shared pathophysiology of GDM and T2DM, i.e., beta cell dysfunction in the setting of insulin resistance. In addition to the risk for T2DM, GDM is also associated with a greater lifetime risk of developing cardiovascular disease [1,2,20,21,22,23,24,25,26]. A recent meta-analysis that included over 5 million women demonstrated that GDM was associated with an approximately two-fold higher risk of developing cardiovascular disease [22]. Importantly, even among women who did not progress to T2DM, GDM was associated with a 1.56-fold relative risk of developing cardiovascular disease, demonstrating that the higher risk of cardiovascular disease does not solely reflect complications associated with T2DM [22].

Recognition of the postpartum risk for cardiometabolic disease among women with a history of GDM provides an opportunity for early preventive interventions to delay or prevent progression to T2DM and/or cardiovascular disease. Indeed, the Diabetes Prevention Program and other studies have demonstrated that pharmacologic and/or lifestyle interventions reduce progression to T2DM in women with a history of GDM [2,27,28,29,30,31]. However, follow-up rates after GDM to assess for progression to T2DM are low [30,32,33,34,35]. The ADA recommends that women with a history of GDM undergo an initial follow-up OGTT 6–12 weeks postpartum followed by lifelong screening for diabetes or prediabetes every 3 years thereafter [36]. Despite this recommendation, follow-up rates with either an OGTT or fasting plasma glucose (FPG) alone in the first 6 months postpartum ranged between 20–60% in different studies [30,32,33,34,35]. 

The reasons for low follow-up rates are multi-factorial, with both patients and healthcare providers contributing to the low rates of follow-up [2,30]. One key factor is that, although women with a history of GDM recognize GDM as a risk factor for future T2DM, they do not perceive themselves as being at high personal risk [37,38]. Currently, interventions to enhance postpartum evaluations and prevention are time and resource intensive and, thus, unlikely to be scalable [30]. However, the potential benefit of enhanced follow-up rates is enormous. It is estimated that postpartum screening and interventions may prevent ~48,000 cases of T2DM annually and thereby save millions of dollars in direct medical costs [30]. Identifying women at particularly high risk for progression during pregnancy would allow for more specific targeting of resource-intensive interventions. Genetic and epigenetic factors contribute to maternal insulin resistance and beta cell function, which are important not only for the development of GDM but also for the long-term complications associated with GDM. Thus, they may provide an approach to identify women at the highest risk for progression to postpartum cardiometabolic disease. The genetic and epigenetic contributions to GDM and its life course are the focus of this review.

## 2. Genetics of GDM

Several observations have suggested that the underlying genetic architecture of T2DM and GDM is similar. These include the high risk for progression from GDM to T2DM, the risk for GDM among women with a family history of T2DM, clustering of T2DM and impaired glucose tolerance in families of women with a history of GDM, and similarity of the pathophysiology underlying GDM and T2DM [1,2,39]. The two primary approaches that have been used to define the genetic architecture of GDM and determine its similarity to that of T2DM are candidate gene analyses and genome-wide association studies (GWAS). 

### 2.1. Candidate Gene Studies

The approach used most frequently to identify genetic variants associated with GDM is candidate gene analysis. In this approach, genetic variants in genes predicted to be related to the pathophysiology of GDM, i.e., candidate genes, are tested. The use of this directed approach has limitations. First, GWAS have demonstrated that many susceptibility genes associated with complex diseases and traits are not obvious strong biological candidates. Second, as the genetic architecture of complex traits and diseases has been further elucidated, it has become apparent that susceptibility genes associated with complex traits and diseases typically have a small effect on disease risk. This has necessitated the use of large sample sizes to achieve sufficient power to identify associated genetic loci. 

Early candidate genes for GDM were often chosen based largely on biological plausibility. These early studies have been the subject of previous reviews [40,41]. Many of these early candidate gene studies contained a relatively small number of women in the control and GDM groups. Thus, a robust and reproducible association of many of the genetic variants tested was not demonstrated. As the results of GWAS for T2DM became available, candidate gene studies for GDM began to focus on genetic variants associated with T2DM [42,43,44]. Many of these variants showed evidence for association with GDM; however, these studies frequently included a limited number of women, so results across studies have been inconsistent. More recently, a meta-analysis of associations reported in 23 studies performed in women of European, East Asian, and South Asian ancestry as well as populations from Latin America and the Middle East was performed [44]. A total of 502 variants were tested with an overall effective sample size ranging from 2373 to 24,237 for the different variants. Sixteen variants in eight genetic loci were significantly associated with GDM. These genetic loci included *IGF2BP2*, *CDKAL1*, *GLIS3*; *CDKN2A/2B*, *HHEX/IDE*, *TCF7L2*, *MTNR1B*, and *HNF1A* (Table 1). Importantly, in addition to being associated with GDM and T2DM, variants in these loci are also associated with measures of beta cell function [44].

### 2.2. Genome-Wide Analysis Studies

A second approach that has been used to identify genetic variation associated with GDM is GWAS. A limitation of candidate gene studies of GDM is that the candidate genes were chosen based upon their biologic plausibility and/or known association with T2DM. GWAS interrogate genetic variants for association with a trait or disease across the whole genome in an unbiased, hypothesis-free manner. Thus, this approach not only validates genetic variants identified through candidate gene studies but also identifies genetic variation uniquely associated with GDM. To date, four GWAS for GDM have been performed. One of these had limited power due to a small cohort and failed to identify any variants with genome-wide significant association (defined as *p* < 5 × 10^−8^ to correct for multiple testing of millions of variants) with GDM [45].

The first GWAS for GDM was performed in a cohort of South Korean women that included 468 cases and 1242 controls in the discovery cohort and 931 cases and 783 controls in a replication cohort [46]. In a joint meta-analysis of the results from the two cohorts, SNPs in *CDKAL1* and *MTNR1B* demonstrated genome-wide significant association with GDM, while *IGF2BP2* demonstrated a near genome-wide significant association. All of these are known T2DM susceptibility genes [47]. A more recent GWAS performed in a multi-ancestry cohort that included 5485 women with GDM and 347,856 without GDM identified five genetic loci associated with GDM. These included *MTNR1B*, *CDKAL1*, *TCF7L2*, *CDKN2A-CDKN2B* and *HKDC1* [48]. The first four loci are also associated with T2DM, while, as described in more detail below, HKDC1 was previously associated with maternal glycemia during pregnancy [49]. A final, recent GWAS performed in a Chinese population that included 193 women with GDM and 819 controls failed to identify any SNPs significantly associated with GDM [50]. Fourteen regions that contained copy number variants (CNVs) were significantly associated with GDM, but none of these were in genetic regions previously associated with GDM.

Both the candidate gene studies and GWAS have demonstrated an overlap between the underlying genetic architecture of GDM and T2DM, consistent with the similar pathophysiology of the two diseases. However, two differences of note have been identified: (i) a greater effect size of variants in *MTNR1B* for GDM compared to T2DM, and (ii) the association of *HKDC1* with GDM but not T2DM. In the GWAS for GDM performed in Korean women, the effect size of the lead SNP in *MTNR1B* was considerably higher in women with GDM (odds ratio = 1.47) compared to East Asian individuals with T2DM (odds ratio = 1.04) [46]. The recent multi-ancestry GWAS for GDM demonstrated a similar finding, with an effect size of the lead SNP in *MTNR1B* for GDM (odds ratio = 1.41) vs. the effect size in a multi-ancestry cohort of T2DM (odds ratio = 1.09) [48]. The multi-ancestry GWAS for GDM also showed a significant association of variants in *HKDC1* with GDM [48]. A prior GWAS in a large multi-ancestry cohort that included over 180,000 individuals with T2DM demonstrated only weak statistical evidence for the association of *HKDC1* with T2DM [47]. 

The mechanism for the greater effect size of variants in *MTNR1B* in GDM compared to T2DM has not been determined. Melatonin is a neuroendocrine hormone that is synthesized primarily in the pineal gland through a series of enzymatic steps that begins with the hydroxylation of tryptophan [51]. It plays an important role in circadian rhythms and sleep but also has important metabolic effects [51]. Studies in human islets suggest that melatonin stimulates the secretion of glucagon and insulin from α- and β-cells, respectively [51]. The metabolic and other effects of melatonin are mediated through binding to transmembrane G-protein-coupled receptors, MT_1_ and MT_2_, the latter of which is encoded by *MTNR1B* [51]. Genetic variants in *MTNR1B* have been associated not only with T2DM but also with several metabolic traits, including fasting glucose, hemoglobin A1c, early insulin response to oral and intravenous glucose, and faster deterioration of insulin secretion over time [52,53,54,55]. Variants in *MTNR1B* are also associated with fasting and 1-h post-load glucose in pregnancy [49].

In addition to its role in regulating circadian rhythms, melatonin is a potent antioxidant capable of removing reactive oxygen species, which may be important during pregnancy [56]. Prior studies in pregnant cohorts demonstrated that circulating melatonin levels increase in the third trimester of pregnancy compared to levels in early pregnancy and non-pregnant women [57,58,59]. Some studies have found higher levels in pregnant women in the first two trimesters of pregnancy vs. non-pregnant controls while others have found similar levels [57,58,59]. It has been suggested that the placenta may be an additional source of melatonin during pregnancy, as serum melatonin levels fell 24 h following a C-section and melatonin was present in the placentas from these women [59]. Indeed, a potential autocrine/paracrine role for melatonin in the placenta to help maintain a balance between villous cytotrophoblasts and the syncytiotrophoblasts has been suggested [56]. If and how these actions of melatonin contribute to maternal glucose metabolism during pregnancy is not known.

*HKDC1* encodes hexokinase domain containing 1 and is a member of the hexokinase family [60]. Initially, a strong association of *HKDC1* with 2 h glucose levels during an OGTT in women at ~28 weeks gestation was demonstrated [49]. In contrast, the lead SNP in *HKDC1* associated with 2 h glucose levels in pregnancy was only nominally associated with 2 h glucose in a large meta-analysis of non-pregnant individuals [61]. Subsequent studies demonstrated that hexokinase domain containing 1 has hexokinase activity and that lower levels of *HKDC1* expression are associated with higher levels of maternal 2 h glucose [62]. The importance of *HKDC1* during pregnancy was further demonstrated using a murine model. Glucose levels were higher in pregnant 8–12 weeks old mice heterozygous for a null mutation of *HKDC1* compared to wild-type mice following an oral glucose challenge at day 15 of gestation, which is a time point of maximal insulin resistance in pregnant mice [63]. In contrast, glucose levels were the same following a glucose challenge in non-pregnant heterozygous and wild-type 8–12 weeks old female mice [63]. Together, these studies suggest that the genetic architecture underlying maternal metabolism during pregnancy has some unique features and that *HKDC1* has a more important role in glucose metabolism during pregnancy than outside of pregnancy.

Given the similarities in the genetic architecture of GDM and T2DM, the ability of genetics to enhance early prediction of women who will develop GDM or women with GDM who will progress to T2DM has been examined. As described above, early identification of women at high risk for progression to T2DM would allow for targeted preventive interventions. Most variants associated with complex diseases, including GDM and T2DM, have a small effect size; thus, carrying any single susceptibility variant confers only minimal risk. To address this, an approach that has been employed is to use aggregate information from multiple susceptibility variants to create a genetic risk score. The association of the genetic risk score with the development of GDM or progression to T2DM can then be examined [42,44,64]. Genetic risk scores that included anywhere from 4 to 84 variants have been associated with GDM in cohorts that included 458 to 2636 cases of GDM and 487 to 13,400 controls [64,65,66,67]. When women were stratified by quantiles or quintiles of genetic risk score, ORs of 1.53 to 3.60 for women in the highest strata of genetic risk score were demonstrated [64,66,67]. One study demonstrated that second to a family history of diabetes, a genetic risk score consisting of only four variants was the most effective predictor of GDM [65]. Another study showed that despite being associated with a high OR for developing GDM, the area under the receiver operating characteristic (ROC) curve for the genetic risk score was 0.64 [64], although another reported that the area under the ROC curve increased from 0.67 to 0.70 with the addition of a genetic risk score to clinical factors [67].

To date, genetic risk scores have had limited utility in predicting T2DM in non-pregnant cohorts. In general, a genetic risk score was of similar efficacy to determining the presence of a family history of T2DM [68]. A limited number of studies have examined the ability of a genetic risk score to predict progression to T2DM in women with a history of GDM. Most, but not all, studies demonstrated the association of a genetic risk score with progression to T2DM [69,70,71,72,73]. The genetic risk scores that have been tested included 13 to 59 SNPs associated with T2DM. One study in women of European ancestry that included maternal age and BMI as predictive factors showed an increase in the area under the ROC curve from 0.627 with clinical factors alone to 0.667 after the addition of a genetic risk score to the model [69]. In a study of Korean women, the addition of a genetic risk score to a model that included maternal age, BMI, and family history of diabetes increased the area under the ROC curve from 0.652 to 0.708 [71]. A more modest increase in the area under the curve was seen when fasting glucose and insulin were included in the model. Thus, to date, only a few studies have addressed whether genetic risk scores can enhance the prediction of progression to T2DM among women with a history of GDM. Whether genetic risk scores that include more SNPs or are more tailored to women with a history of GDM will be efficacious in enhancing prediction above clinical factors alone will need to be determined in future studies as the genetic architecture of GDM is better defined.

A final approach that can be used to further characterize GDM using genetics is soft clustering. Clustering involves dividing a population of data points into groups such that data points in the same group are more similar to other data points within that group than to data points in other groups. Put simply, the goal of clustering is to segregate groups with similar traits and assign them to clusters. In soft clustering, data points are not assigned to separate clusters, rather a probability or likelihood of a data point being in a cluster is assigned [74]. This approach has been applied to T2DM to better define T2DM heterogeneity. Using GWAS data and diabetes-related traits, five clusters of T2DM loci and traits were identified [75]. Two clusters included variant-trait associations characteristic of decreased beta cell function and were differentiated by high (beta cell cluster) vs. low proinsulin levels (proinsulin cluster). The other three clusters were more characteristic of insulin resistance and included obesity-mediated (high BMI and waist circumference), lipodystrophy-like fat distribution (low BMI, adiponectin, and high-density lipoprotein cholesterol, and high triglycerides), and disrupted liver lipid metabolism (low triglycerides) clusters.

This approach has been applied recently to GDM in a study that included more than 5000 women in a multi-ancestry cohort [76]. Initial studies examined the association of the five T2DM clusters with GDM. GDM was not associated with the beta cell cluster in the full multi-ancestry cohort, but this cluster was associated with GDM in women of European ancestry. The liver-lipid cluster was associated with GDM in the full multi-ancestry cohort, while the other three clusters were not associated with GDM. In a second approach, a subset of the cohort was used to derive five pregnancy-related clusters that were characterized by different glycemic traits and genetic variants [76]. One cluster that was characterized by postload glucose levels, lower disposition index, and higher adiposity measures was associated with GDM. None of the other four clusters was associated with GDM. When the association of these pregnancy clusters was tested in a cohort of over 30,000 individuals, 4910 of whom had T2DM, three of the clusters were associated with T2DM, including the cluster associated with GDM. Of interest, the effect size for the association of this cluster with GDM was greater than the effect size of its association with T2DM (odds ratio for GDM 1.24 vs. 1.11 for type 2 diabetes). Consistent with the findings with *MTNR1B* and *HKDC1*, these findings suggest that the genetic and physiologic pathways leading to GDM may differ, in part, from those leading to T2DM.

### 2.3. Transcriptome and GDM

Genetic variation associated with complex diseases such as GDM and phenotypes such as glucose metabolism during pregnancy typically results in changes in gene expression that contribute to disease risk and/or phenotypic differences [77]. To that end, a few studies have reported GDM-related differences in the transcriptome of blood cells [78]. Steyn et al. and Zhao et al. used RNA-seq and a microarray platform, respectively, to obtain gene expression profiles of blood cells in women with GDM compared to normoglycemic controls [79,80].

Steyn et al. performed RNA-seq using blood cells collected at 29–33 weeks gestation and identified 1008 differentially-expressed genes in women with GDM vs. normal glucose tolerance [79]. Gene set enrichment analysis demonstrated the enrichment of carbohydrate and NADPH metabolic pathways with the notable clustering of five genes encoding enzymes in the pentose phosphate pathway. The mRNA level of one of these genes, *G6PD* (which encodes glucose 6-phosphate dehydrogenase), was inversely associated with levels of fasting, 1 h, and 2 h glucose levels. Using a microarray-based approach, Zhao et al. identified 2709 genes with greater expression and 2488 genes with lower expression in blood cells from women with GDM at the time of screening for GDM [80]. Subsequent gene ontology term and pathway analyses using these mRNAs suggested that immune and inflammatory functional categories were important for the pathogenesis of GDM. This is consistent with a recent study demonstrating higher expression of inflammation-related genes in blood cells from women with GDM compared to women with normal glucose tolerance [81] and earlier studies suggesting an important role for inflammation in GDM [82]. 

## 3. GDM and Epigenetics

Epigenetics is defined as heritable changes in gene expression that are not due to changes in the sequence of DNA [83,84,85]. The primary types of epigenetic modifications include DNA methylation, modifications of histones (acetylation, methylation, phosphorylation, and ubiquitination), and non-coding RNAs. The epigenetic mechanism most studied to date is DNA methylation. DNA methylation involves the addition of a methyl group to the 5 position of cytosine residues to form 5-methylcytosine, a process catalyzed by DNA methyltransferase [86]. DNA methylation typically occurs on a cytosine that precedes a guanine nucleotide (CpG), with clusters of these CpG sites forming CpG islands. However, DNA methylation also occurs at non-CpG sites. CpG islands are typically, but not exclusively, located within gene promoters, with hypermethylation of a promoter being associated with decreased gene expression and hypomethylation increased gene expression. DNA methylation often occurs in response to changes in an organism’s environment [85]. Histone modifications are important for the regulation of chromatin structure and gene expression [87,88]. Depending on the type and location of the modification, they result in structural changes in chromatin that suppress or promote gene expression. Acetylation of histones typically is associated with active chromatin and gene transcription [89], while histone methylation is associated with both activation and repression of gene transcription [90]. A number of classes of non-coding RNAs have been identified. One that has been examined in GDM and is associated with changes in gene expression is microRNAs (miRNAs). miRNAs regulate gene expression at the post-transcriptional level by binding to the 3′ untranslated region and inhibiting the translation of and/or inducing degradation of their target mRNAs [91]. In some cases, a single miRNA can regulate the expression of a large number of proteins [92,93]. An additional class of non-coding RNAs is the long non-coding RNAs, which have a variety of different functions [94,95,96].

Epigenetics can be used to characterize and predict GDM as well as its long-term outcomes. By doing so, it can identify potential biomarkers for GDM and its associated outcomes. Biomarkers that are most feasible for translation to clinical care are those that are accessible through non- or minimally invasive approaches and are stably expressed in biological fluids [97]. The major focus of epigenetic studies in GDM has been on the placenta and cord blood to identify markers associated with offspring outcomes [98]. The focus of this review is on gestational diabetes and maternal outcomes and will, therefore, review studies of epigenetic changes in maternal blood, serum, and plasma. 

### 3.1. DNA Methylation

Most studies examining the role of epigenetic changes in GDM have focused on DNA methylation. Similar to genetic studies, two different approaches have been used to examine DNA methylation, targeted studies of sites within candidate genes and epigenome-wide association studies (eWAS). Similar to GWAS, the latter approach is an unbiased approach to identifying novel sites associated with a trait or disorder of interest. Studies have been performed using samples at three time points: (i) early in pregnancy to assist with the prediction of women who will develop GDM, (ii) at the time of diagnostic testing for GDM, and (iii) at delivery.

A limited number of studies have examined DNA methylation in blood cells collected from women early in pregnancy (less than 20 weeks gestation) [99,100,101]. Two studies performed an eWAS, but both were small (11 and 6 cases with the same number of women with normoglycemia during pregnancy). Wu et al. identified 100 differentially methylated sites in GDM compared to normoglycemic women, with the majority of these being hypomethylated in women with GDM [101]. Two of these sites were in Hook Microtubule-Tethering Protein 2 (*HOOK2*) and Retinol Dehydrogenase 12 (*RDH12*), which had been identified in an earlier study that examined GDM-related changes in DNA methylation in cord blood and placenta [102]. A second study examined DNA methylation in blood cells collected from the same women during two different pregnancies, with one pregnancy complicated by GDM and the other not [99]. Twenty-seven differentially methylated CpG sites (17 hypomethylated and 10 hypermethylated) were identified in the GDM-complicated pregnancy compared to the pregnancy with normoglycemia. One of the hypomethylated sites was in *CDKN2B*, which is within a genetic locus associated with T2DM and encodes cyclin-dependent kinase inhibitor 2B. Finally, Wang et al. examined 337 CpG sites in peripheral blood collected at 10–15 weeks gestation from 80 women with GDM and 80 normoglycemic women [100]. The 337 sites were chosen based on findings from earlier studies. Methylation levels in women with GDM were higher at six of the sites and lower at six sites. When the utility of these sites for the prediction of GDM was tested, the area under the ROC curve ranged from 0.590 to 0.653. The ability of the methylation status of these sites to improve prediction beyond clinical factors alone was not tested. In this same study, the methylation statuses of CpG sites in *HAPLN3*, *RDH12*, *DNAJB6*, and *NFATC4* were significantly associated with the development of GDM [100]. 

Three studies examined DNA methylation in maternal blood at the time of the diagnostic OGTT for GDM [79,103,104]. One study demonstrated that global DNA methylation in blood did not differ between women with and without GDM [104]. This same group performed an eWAS using DNA prepared from the blood of 12 women diagnosed with GDM compared to 12 normoglycemic women [103]. They identified 1046 differentially methylated sites with 14.2% hypermethylated and 85.8% hypomethylated in women with GDM. A final study used blood cells to examine DNA methylation in the promoters of five genes that were differentially expressed in six women with GDM compared to six normoglycemic women [79]. These five genes encoded glucose-6-phosphate dehydrogenase, transketolase, and insulin growth factor binding protein-1, -2, and -6. Higher methylation of a CpG site in the *G6PD* promoter was observed in GDM compared to normoglycemic women; no differences were observed in CpG sites in the other genes.

A last group of studies examined DNA methylation at the time of delivery [105,106,107]. Canouil et al. performed an eWAS in blood collected from 536 mother-newborn pairs, 298 of whom had GDM [105]. They sought to identify differentially methylated regions that were shared between the mother and newborns. After adjusting for newborn sex, birth weight, and gestational age and maternal age, gestational weight gain, and pre-pregnancy BMI, no differentially methylated CpG sites associated with GDM exposure were identified. Another eWAS was performed in DNA from the blood of eight women with GDM and eight controls [106]. Included among the 150 genes that contained the top 200 differentially methylated regions were the genes encoding interleukin-6 and interleukin-10. This is consistent with another study from this group that was performed in 8 women with GDM and 25 controls and demonstrated hypomethylation of CpG sites in *IL10* and higher circulating levels of interleukin-10 in women with GDM [108]. A final study examined methylation of CpG sites in the promoter of *INSR*, which encodes the insulin receptor, in DNA in blood from 25 GDM and 30 normoglycemic women [107]. Hypermethylation in the *INSR* promoter of GDM compared to normoglycemic women was observed.

More recently, the ability of differentially methylated CpG sites to aid in the prediction of women who will progress to abnormal glucose tolerance in the post-partum period was reported [109]. An eWAS was performed in a discovery cohort of 24 women with GDM and 24 controls using DNA from blood collected between 26 and 30 weeks of gestation. No differentially methylated CpG sites that were statistically significant after correction for multiple testing were found, but 50 sites had a nominal *p* value between 10^−4^ and 10^−5^, and the top 7 differentially methylated sites were examined in a replication cohort. After adjusting for maternal age, BMI, and gestational weight gain, significant hypermethylation in CpG sites in *LINC00917*, a long non-coding RNA, and *CTBP2*, which encodes C-terminal binding protein 2, was found in women with GDM. One CpG site in *LEF1*, which encodes lymphoid enhancer binding protein 1, was hypomethylated in the discovery cohort but hypermethylated in the replication cohort. None of the other four sites tested exhibited a significant difference in methylation in the replication cohort. Seventy-nine of the women with GDM in the replication cohort returned for a post-partum evaluation of their glucose tolerance, 27 of whom had abnormal glucose tolerance (prediabetes or diabetes). The other 54 women had normal glucose tolerance. After adjustment for maternal pregestational BMI, age, and gestational weight gain, a significant difference in methylation was observed at CpG sites in *LINC00917* and *TRAPPC9*, which encodes trafficking protein particle complex subunit 9, in the women with abnormal glucose tolerance. When the methylation status at these three sites was added to a model that included clinical factors, the area under the ROC curve for prediction of progression to abnormal glucose tolerance postpartum increased from 0.760 to 0.853, although this increase did not reach statistical significance.

Overall, these studies suggest that there are differences in DNA methylation in the blood of women with GDM compared to normoglycemia and that in some cases these differences are evident in the first or early second trimester (Table 2). However, to date, most of these studies have been small, did not include validation studies, and, in some cases, did not adjust for a robust group of covariates. A recent study that included both a discovery and replication cohort suggested that methylation status during pregnancy may be able to contribute to the prediction of women who will progress to abnormal glucose tolerance post-partum (Table 2). Future studies with larger cohorts or consortia that include the results from multiple cohorts will be needed to more clearly define changes in DNA methylation characteristic of GDM and/or predict GDM or progression to abnormal glucose tolerance post-partum.

### 3.2. Histone Modification

Examination of histone modifications in GDM has been limited. Michalcyzk et al. measured levels of lysine dimethylation in five histones, H3K27, H3K4, H3K79, H3K36, and H3K9, in peripheral blood. Samples were collected in the third trimester (30 weeks gestation) and in the early postpartum period (8–10 weeks and 20 weeks) from pregnant women with normal glucose tolerance and in pregnant women with GDM who did and did not develop T2DM postpartum [110]. At 30 weeks gestation, lysine dimethylation of H3K79 and H3K36 was lower in women with GDM, in both those who did and did not progress to T2DM postpartum, compared to women with normal glucose tolerance in pregnancy. However, there was no difference in lysine dimethylation of any of the five histones between the two groups with GDM. At 8–10 weeks postpartum, H3K4 lysine dimethylation was lower in women with GDM who developed T2DM compared to women with GDM who did not develop T2DM. Lysine dimethylation of the other histones did not differ between the two groups at 8–10 weeks postpartum. Finally, at 20 weeks postpartum, H3K27 dimethylation was lower and H3K79 dimethylation was higher in women with GDM who developed T2DM compared to women who did not develop T2DM, while lysine dimethylation levels of the other three histones did not differ between the two groups. This study demonstrated that lysine dimethylation differed in the postpartum period between women with GDM who did and did not develop T2DM, but consistent changes in dimethylation were not observed. In addition, differences in dimethylation were not present between the groups with GDM at 30 weeks gestation. Importantly, the period of postpartum follow-up was short and more women with GDM would likely progress to T2DM over time. Thus, the role of histone modifications in helping with the prediction of women who will develop GDM and/or T2DM postpartum awaits further studies with larger cohorts. 

### 3.3. Non-Coding RNAs

Non-coding RNAs account for the majority of the transcriptional output from the genome and are involved in a variety of cellular processes [111,112]. Non-coding RNAs are generally divided into two groups, long non-coding RNAs, which are defined as non-coding RNAs over 200 nucleotides in length, and small non-coding RNAs that are under 200 nucleotides in length [111,112]. The best characterized among the latter group are microRNAs (miRNAs). Over the last several years, a potential role for non-coding RNAs in GDM has been examined.

#### 3.3.1. microRNAs

MicroRNAs are involved in a wide range of cellular and physiological processes, including glucose homeostasis and insulin secretion [111]. Given their stability in blood and other body fluids, the utility of miRNAs as non-invasive biomarkers has been examined, including for GDM. An increasing area of interest is identifying circulating miRNAs that are present early in pregnancy and associated with the development of GDM and using these miRNAs to help predict the development of GDM [113,114,115,116,117,118,119,120,121,122]. To date, many of the studies that have been performed have been small with limited power and have used different approaches to identify relevant miRNAs. These approaches have included targeting specific miRNAs, using various platforms that screen for pre-determined subsets of miRNAs, and using an unbiased sequencing-based approach to identify miRNAs associated with the phenotype being examined. Given these varying approaches, only a limited number of miRNAs have been identified in more than one study, but the results to date demonstrate the potential utility of including miRNAs in models to predict GDM.

Together, studies seeking to identify miRNAs in the first trimester and early second trimester of pregnancy have identified 37 miRNAs associated with the development of GDM (Table 3) [113,114,115,116,117,118,119,120,121,122]. However, most of these miRNAs were specific to a single study and not reproduced in other studies. One miRNA of interest is miR-16-5p, for which higher levels in women going on to develop GDM were demonstrated in four different studies [113,116,117,121]. The target genes for miR-16-5p include the genes encoding the insulin receptor and insulin receptor substrate-1 and -2, which are important for insulin signaling, as well as genes important for β-cell proliferation and apoptosis [116]. A second miRNA associated with GDM in two studies was miR-17-5p [113,121]. Higher levels of miR-20a-5p in early pregnancy were also associated with GDM in two studies [113,121], but in a third cohort of South African women, lower levels of miR-20a-5p were found in women who developed GDM [123]. One target of miR-20a-5p is insulin receptor substrate-1 [124].

Circulating miRNAs present in the first and early second trimester have demonstrated the potential utility of miRNAs in predicting who will progress to GDM, as shown by their inclusion in models for prediction of GDM and reflected by the area under the ROC curve [115,116,117,119]. Juchnika et al. used age-, BMI-, and gestational age-matched women who progressed to GDM or maintained normoglycemia to demonstrate that each of three miRNAs, miR-16-5p, -144-3p, and 142-3p, resulted in an area under the ROC curve between 0.756 to 0.868 [116]. Given that the women in the two cohorts were matched, clinical predictors were not included in the model. Sorenson et al. demonstrated that the incorporation of three miRNAs, miR-16-5p, 29a-3p, and -134-5p, into a predictive model that included fasting blood glucose in the first trimester increased the area under the ROC curve from 0.756 with fasting glucose alone to 0.868 when the miRNAs were included [117]. Finally, Legare et al. included three miRNAs, miR-517a-3p/517b-3p, -218-3p, and -7a-3p, in a predictive model that included maternal age, BMI, family history of type 2 diabetes, history of GDM, and hemoglobin A1c. The inclusion of the miRNAs increased the area under the ROC curve from 0.754 to 0.841 [115]. Importantly, these results were validated in an independent cohort. Finally, in a cohort of 421 women, 55 of whom developed GDM, next-generation sequencing was used to quantify miRNAs in plasma obtained at 4–16 weeks gestation [114]. Thirty-nine miRNAs were associated with insulin sensitivity, 18 of which independently predicted insulin sensitivity at ~26 weeks gestation. Pathway analysis suggested that these miRNAs were important for fatty acid biosynthesis and metabolism. 

MicroRNAs associated with GDM at the time of diagnostic testing for GDM have also been identified [125,126,127,128]. These miRNAs were identified using relatively small cohorts that screened for a varying number of miRNAs. Three of the studies identified one miRNA associated with GDM, while one study identified three associated miRNAs. Two of the studies identified miR-330-3p as being associated with GDM [126,127]. Among the pathways predicted from the gene targets of miR-330-3p is insulin signaling.

Finally, miRNAs have been examined as potential biomarkers for progression to T2DM or the development of cardiovascular disease in women with a history of GDM [129,130]. Joglekar et al. measured miRNAs in a cohort of 103 women with GDM in blood collected 12 weeks postpartum and followed the cohort for up to 10 years [130]. In total, 754 miRNAs were screened in a discovery cohort, and 15 of these were measured in a validation cohort. One of the miRNAs, miR-369-3p, was significantly associated with progression to T2DM in a fully adjusted model. The predictive ability of miR-369-3p was tested by adding it to a model that included six clinical factors, including maternal age, BMI, fasting glucose in pregnancy and postpartum, cholesterol, and triglycerides. The inclusion of miR-369-3p increased the area under the ROC curve from 0.83 to 0.92. A second study examined the prediction of developing cardiovascular disease in women with a history of GDM using miRNAs [129]. Twenty-nine miRNAs known to be involved with insulin resistance, hypertension, or cardiovascular disease were screened 3 to 11 years postpartum in women with a history of GDM or normoglycemic control women. Of the miRNAs that were screened, 26 were present at higher levels in women with a history of GDM. When 16 of these miRNAs were included in a model to predict mothers who will develop cardiovascular or cerebrovascular disease postpartum, the area under the ROC curve was 0.900. Together, these studies demonstrate that miRNAs, in addition to assisting with the prediction of GDM, may also be useful for predicting the long-term adverse outcomes associated with GDM.

#### 3.3.2. Long Non-Coding RNAs

Long non-coding RNAs (lncRNAs) are defined as RNAs greater than 200 nucleotides in length that are not transcribed into functional proteins [94,95,96]. The number of lncRNAs estimated to be present in the human genome has ranged from 16,000 to over 100,000 [94,95,96]. While not all lncRNAs are functional, roles for lncRNAs in genome organization, cell structure, gene expression, and physiological processes have been described [94,95,96]. Regulation of gene expression by lncRNAs occurs at multiple levels, including at the transcriptional, post-transcriptional, translational, and post-translational levels [94,95,96,131]. Given their broad role in cellular and organismal physiology, the contribution of lncRNAs to disease states has been described [94,95,96,131], including recent studies examining a potential role for lncRNAs in GDM [111].

Similar to the approach used for other epigenetic changes, approaches to examine changes in a broad range of lncRNAs as well as specific lncRNAs in GDM have been examined. Li et al. used microarray analyses to examine the levels of a broad range of lncRNAs in plasma collected between 24 and 40 weeks gestation from 3 women without and with GDM. Increased expression of 609 and decreased expression of 689 lncRNAs were found in plasma from women with GDM [132,133]. Subsequent network analyses identified six lncRNAs together with four mRNAs that played a role in insulin resistance [133]. Differential expression of four of these six lncRNAs was demonstrated in a small cohort of women without and with GDM. A second study that used an array-based approach with blood collected early in pregnancy from 3 women without and with GDM demonstrated higher expression of 197 lncRNAs in women with GDM [134]. Finally, Fu et al. examined lncRNA-mediated feed-forward loops in which an mRNA and miRNA coordinate to regulate the expression of a lncRNA [135]. Using blood collected at 24–32 weeks gestation from 8 women without and with GDM, a global lncRNA feed-forward loop network was created from which glycometabolism and hormone-related lncRNA feed-forward loop networks were extracted. Consistent with a role for lncRNAs in GDM, 11 glycometabolism and 29 hormone-related lncRNA feed-forward loop networks were found to be dysregulated in GDM. 

Complementing these array-based approaches are studies examining the role of specific lncRNAs in GDM. Studies to date are relatively limited with only one lncRNA being examined in more than one study. Zhang et al. initially examined the lncRNA MEG8 in GDM based on previous studies, demonstrating that its expression could be induced by high glucose [136]. They measured MEG8 levels prior to pregnancy in plasma from 400 women, 78 of whom developed GDM. Women who subsequently developed GDM had higher levels of MEG8, and higher pre-pregnancy levels of MEG8 were associated with a higher incidence of GDM. A subsequent study demonstrated that pregnant women with GDM had higher blood levels of MEG8 compared to women with normal glucose tolerance [137]. Other studies examined levels of specific lncRNAs which earlier studies had suggested were associated with T2DM or glucose-related traits. Higher blood levels of five lncRNAs, including HOTAIR, XIST, MALAT1, RPL13P5, and MEG3, and lower levels of SNH617 and DANCR were found in women with GDM [132,138,139,140,141,142,143]. In addition, lower levels of SNHG17 and higher levels of MEG8 predicted GDM ~1 month before the diagnosis of GDM with an area under the ROC curve of 0.72 to 0.74 [136,138]. Finally, associations of lncRNAs with various metabolic traits have been observed. These include a positive association of HOTAIR with maternal BMI and fasting, 1 h, and 2 h glucose, of RPL13P5 with insulin resistance, and of XIST with fasting glucose, and an inverse association of DANCR with blood glucose levels [132,139,140,141].

Together, these studies suggest that lncRNAs may contribute to the pathogenesis of GDM, although the mechanisms underlying the observed associations have yet to be demonstrated. To date, the association or role of lncRNAs in the long-term metabolic outcomes associated with GDM has not been examined.

#### 3.3.3. Circular RNAs

A final group of non-coding RNAs is the circular RNAs (circRNAs), which are produced by backsplicing of RNA [144]. This occurs when a downstream splice-donor site covalently links to an upstream splice-acceptor site. More than 100,000 circRNAs have been identified, although the function of only a limited number of circRNAs has been defined [144]. Roles for circRNAs that have been described include serving as an inhibitor of miRNAs or proteins or regulating protein function [144]. Despite their inclusion with non-coding RNAs, some circRNAs are translated [144]. Consistent with the limited studies on circRNAs, only a few studies have examined their role in GDM. After using RNA sequencing to demonstrate lower placental levels of the circRNA hsa-circ-000243 in the placentas of women with GDM, Wang et al. demonstrated that plasma levels of hsa-circRNA-0005243 were lower in women with GDM compared to controls at 37–40 weeks gestation [145,146]. Based on an earlier study that demonstrated up- and downregulation of multiple circRNAs in the placentas of women with GDM [147], Wu et al. examined the levels of six circRNAs in the serum of women with GDM and normoglycemic controls during the second and third trimesters of pregnancy [148]. They demonstrated higher levels of hsa-circRNA-0054633 in women with GDM during the second trimester of pregnancy together with a positive correlation with the levels of hemoglobin A1c and 2-hr glucose during an OGTT. Serum levels of hsa-circRNA-0054633 remained higher in women with GDM during the third trimester. While much remains to be learned about the role of circRNAs in GDM, the limited studies to date are consistent with a potential role for this class of non-coding RNAs in GDM.

### 3.4. Small Extracellular Vesicles

Extracellular vesicles (EVs) are secreted vesicles that are delimited by a lipid bilayer and contain bioactive molecules, including proteins, mRNAs, and miRNAs [149]. EVs that are produced from the late endosomal pathway and released upon fusion of multivesicular bodies with the plasma membrane were originally referred to as exosomes and are typically ~50 to 150 nm in diameter. Upon recommendation of the International Society of Extracellular Vesicles, this heterogenous population of EVs are referred to as small EVs (sEVs) [149]. sEVs are secreted from most cell types, including the placenta, and can mediate autocrine, paracrine, and endocrine effects based upon their cargo, which can be delivered to other cells and tissues [149].

In a study of plasma collected from women with GDM or normal glucose tolerance during early, mid-, and late pregnancy, the number of circulating sEVs increased across gestation in women with normal glucose tolerance and GDM [150]. In addition, the number of circulating sEVs was higher in women with GDM compared to women with normal glucose tolerance at each time point. The sEVs were able to stimulate cytokine release from human umbilical vein endothelial cells with a larger effect of GDM compared to control sEVs [150]. Two other studies have examined the impact of sEVs isolated from women with GDM compared to normal glucose tolerance during pregnancy on metabolism in mice [151,152]. For these studies, sEVs were isolated from plasma collected from women at 24–28 weeks gestation and infused into mice. When infused into non-pregnant female mice, higher levels of fasting insulin were observed in mice infused with sEVs from women with normal glucose tolerance compared to GDM [151]. In addition, glucose-stimulated insulin secretion was decreased in islets isolated from mice infused with GDM sEVs, and both basal and insulin-stimulated phosphorylation of two insulin signaling molecules, IRS-1 and Akt, were reduced in skeletal muscle strips isolated from mice infused with GDM sEVs [151]. In a related study, sEVs prepared from women with GDM or normal glucose tolerance during pregnancy were infused into pregnant mice for 4 days beginning on day 13.5 of gestation [152]. Following the infusion, higher glucose levels at 30 and 45 min and the area under the glucose curve were observed during an OGTT following infusion of sEVs from women with GDM [152]. In addition, fasting insulin levels were lower in mice infused with sEVs from women with GDM compared to normal glucose tolerance, and decreased glucose-stimulated insulin secretion was observed in islets isolated from mice infused with sEVs from women with GDM [152]. Finally, a recent study using EVs isolated from plasma of pregnant women with normal glucose tolerance or GDM at 24–28 weeks gestation demonstrated that the EVs from women with GDM decreased glucose uptake into a human hepatic cell line [153]. Together, these studies suggest that circulating sEVs in women with GDM contain cargo that impairs insulin secretion and sensitivity and glucose metabolism. 

The cargo present in sEVs includes miRNAs [149]. Three studies have examined miRNAs present in sEVs purified from women with normal glucose tolerance compared to women with GDM [153,154,155]. Gillet et al. screened 17 miRNAs in sEVs isolated from the plasma of women at 6–15 weeks gestation [154]. The levels of 10 miRNAs were higher in plasma from women who subsequently developed GDM compared to women who maintained normal glucose tolerance. The targets of these miRNAs were involved in pathways relevant to GDM, including insulin receptor and AMP-activated protein kinase (AMPK) signaling. In a second study, Nair et al. isolated sEVs from plasma during early, mid-, and late pregnancy and used RNA sequencing to identify miRNAs that were present [155]. Levels of 101 miRNAs were different between sEVs isolated from women with GDM compared to normal glucose tolerance, with six miRNAs varying significantly across gestation in women with GDM compared to normal glucose tolerance. Included among these miRNAs was miR-16-5p, which was shown in studies described above to differ between normal glucose tolerant and GDM women. Finally, Ye et al. used next-generation sequencing to identify miRNAs differentially expressed in sEVs collected at 24–28 weeks gestation from women with GDM or normal glucose tolerance [153]. Differential expression of five miRNAs, miR-423-5a, -122-5p, -148-3p, -192-5p, and -99a-5p, was validated in independent samples. These miRNAs were also associated with various metabolic traits, including maternal pre-pregnancy BMI, hemoglobin A1c, and fasting, 1 h and 2 h glucose levels. Two of these miRNAs, miR-423-5p and -122-5p, had been identified in previous studies of miRNAs in EVs associated with GDM [154,155]. Finally, when these five miRNAs were used at 10–16 weeks gestation to predict the subsequent development of GDM, the area under the ROC curve was 0.82. Interestingly, this was much higher than the area under the curve for clinical factors, and clinical factors did not add to the area under the curve for the miRNAs. Together, these studies demonstrate that EVs contain cargo that impacts glucose metabolism during pregnancy and may contribute to the development of GDM.

The downstream impacts of the genetic and epigenetic changes associated with GDM are changes in the synthesis, stability, and activity of proteins. While beyond the scope of this review, changes in the proteome in GDM have been described and recently reviewed [156,157].

## 4. Conclusions

**Figure 1:**
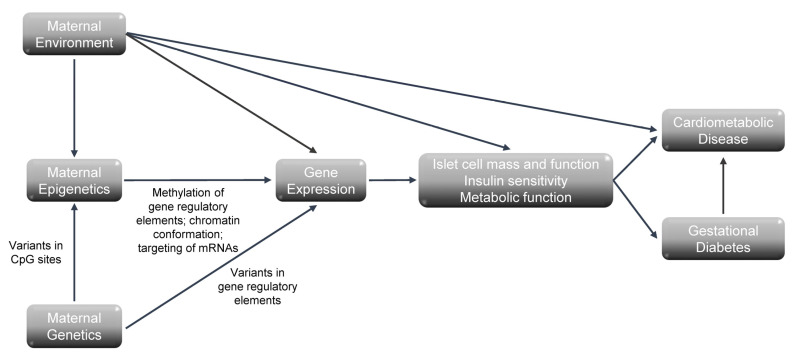
Model for the relationship of maternal genetics, epigenetics, and environment in the risk for gestational diabetes and its long-term cardiometabolic outcomes.

GDM and its impact on the life course is a complex process that involves maternal genetics, epigenetics, the environment, and their interaction (Figure 1). As described, genetic variation, through its effect on gene expression and, ultimately, islet cell mass and function and insulin sensitivity, is associated with risks for both gestational diabetes and T2DM, with an overlap of the genetic variants associated with these two disorders. Epigenetic modifications, which are regulated by both the maternal environment and genetic variation, also impact gene expression and are associated with the risk of GDM and its long-term complications. The role of genetic factors and epigenetic modifications in the long-term risk of cardiovascular disease in women with a history of GDM remains to be explored.

Although much remains to be learned about the interactions of the maternal environment, genetics, and epigenetics in the risk for GDM and its long-term cardiometabolic complications, advances in these areas have provided new insight into the pathophysiology of GDM and its impact on the life course of affected women. This new insight has also allowed for the development and testing of predictive models for the diagnosis of GDM and its long-term outcomes. Robust predictive models have the potential to help target preventive interventions to those most at risk and thereby impact clinical care. However, much remains to be done. Many of the studies to date have been small, and findings have often not been replicated across studies. Going forward, larger cohorts and/or the formation of consortia to increase the power and reproducibility of findings will be needed together with the development of analytic and technical approaches that integrate the various genomic, epigenomic, and other “omics” technologies.

## Figures and Tables

**Table 1:** Genetic loci associated with GDM in recent meta-analysis.

Gene	Chromosome	Encoded Protein	Protein Function
*IGF2BP2*	3	Insulin-like growth factor 2 mRNA-binding protein 2	Binds insulin-like growth factor-2 mRNA and regulates IGF-2 translation; risk allele associated with decreased insulin secretion
*CDKAL1*	6	CDK5 regulatory subunit associated protein 1 like-1	A tRNA methylthiotransferase; function not clearly elucidated but non-pregnant carriers of the risk allele have impaired oral and intravenous glucose-stimulated insulin secretion
*GLIS3*	9	GLIS family zinc finger 3	Zinc finger protein; expressed in the developing pancreas; regulates insulin gene transcription; associated with fasting glucose and beta cell function
*CDKN2A/2B*	9	Cyclin-dependent kinase inhibitor 2A/B	Encode inhibitors of cyclin-dependent kinase; associated with impaired glucose-stimulated insulin secretion
*HHEX/IDE*	10	Hematopoietically expressed homeobox/insulin-degrading enzyme	HHEX encodes a homeobox family transcription factor; IDE encodes insulin-degrading enzyme; associated with decreased insulin response to oral glucose and β-cell glucose sensitivity
*TCF7L2*	10	Transcription factor 7-like 2	Transcription factor and member of the Wnt signaling pathway; risk allele associated with reduced insulin secretion
*MTNR1B*	11	Melatonin receptor 1B	G-protein coupled receptor that is expressed in beta cells; stimulates insulin and glucagon secretion in human islets; associated with fasting glucose and early insulin response to oral and intravenous glucose
*HNF1A*	12	Hepatocyte nuclear factor-1α	Transcription factor required for expression of liver-specific genes; mutations cause maturity-onset diabetes of the young (MODY) 3; associated with altered insulin secretion

**Table 2:** Genetic loci with replicated changes in methylation associated with development of GDM or postpartum type 2 diabetes.

Gene	Chr	Risk Associated with Hypermethylation	Gene Function	Reference
Loci associated with development of GDM				
*DNAJB6* (DnaJ heat shock protein family [Hsp40] member B6)	7	Decreased	Member of the heat shock protein 40 family; acts as a molecular chaperone for cellular processes; relationship to type 2 diabetes not clear	[89,90]
*RDH12* (Retinol dehydrogenase 12)	14	Increased	Key enzyme in metabolism of retinoids; highest activity towards 9-cis and all-trans-retinol which stimulate insulin secretion in a beta cell line	[90,91]
*NFATC4* (Nuclear factor of activated T cells 4)	14	Increased	Component of a DNA-binding transcription complex; promotes release of inflammatory factors from adipose tissue; negatively regulates adiponectin gene expression in fat cells	[90]
*HAPLN3* (Hyaluronan and proteoglycan link protein 3)	15	Decreased	Involved in organization and stability of hyaluronic acid-dependent extracellular matrix; hyaluronic acid is a component of extracellular matrix in islets	[89,90]
Loci associated with development of postpartum type 2 diabetes				
*TRAPPC9* (Trafficking protein particle complex subunit 9)	8	Increased	Plays a role in activation of NF-κB	[100]
*LINC00917* (Long intergenic non-protein coding RNA 917)	16	Increased	Long non-coding RNA; genetic variants in locus associated with obesity in children, lipid-lowering statin response, and diabetic retinopathy	[100]

**Table 3:** MicroRNAs associated with the development of GDM.

Study (Reference No.)	Number of Subjects	Approach for Identification of miRNAs	miRNAs Associated with Risk of GDM
Cao et al. [103]	85 GDM/72 controls	Screened 5 miRNAs	**miR-16-5p**, **-17-5p**, **-20a-5p**
Legare et al. [105]	Discovery: 56 GDM/380 controls Replication 76 GDM/63 controls	Next-generation sequencing	miR-517a-3p/miR-517b-3p, -141-3p, -519c-3p, -520a-3p, -1323, -524-5p, -516b-3p, -218-5p, -429, -516a-5p, -196a-5p, -215-5p, -515-3p, -424-5p, -7a-3p, -525-5p, -518f-5p
Juchnicka et al. [106]	24 GDM/24 controls	Screened 800 miRNAs	**miR-16-5p**, -142-3p, 144-3p
Sorenson et al. [107]	82 GDM/41 controls	Screened 8 miRNAs	**miR-16-5p**, -29a-3p, -134-5p
Wander et al. [108]	36 GDM/80 controls	Screened 10 miRNAs	miR-155-5p, -21-3p
Yoffe et al. [109]	23 GDM/20 controls Replication: 10 GDM/10 controls	Screen 798 miRNAs	miR-223, -23a
Zhao et al. [110]	Discovery: 24 GDM/24 controls Replication: 3 cohorts total 68 GDM/68 controls	TLDA chips (667 miRNAs)	miR-132, -29a, -222
Zhu et al. [111]	Pooled samples 10 GDM/10 controls	High throughput sequencing	**miR-16-5p**, **-17-5p**, -19a-3p, 19b-3p, **-20a-5p**
Lamadrid-Romero [112]	67 GDM/74 controls	Screened 12 miRNAs	miR-183-5p, -200b-3p, 125b-3p, -1290
Pheiffer et al. [113]	28 GDM/53 controls	Screened 8 miRNAs	**miR-20a-5p** *, -222-3p

miRNAs identified in more than one study are bolded; *, lower levels in women with GDM in this study vs. higher levels in 2 earlier studies.

## Data Availability

No new data were created for this article.
